# The evolutionary pattern of mutations in glioblastoma reveals therapy-mediated selection

**DOI:** 10.18632/oncotarget.23541

**Published:** 2017-12-15

**Authors:** Andrea M. Muscat, Nicholas C. Wong, Katharine J. Drummond, Elizabeth M. Algar, Mustafa Khasraw, Roel Verhaak, Kathryn Field, Mark A. Rosenthal, David M. Ashley

**Affiliations:** ^1^ School of Medicine, Deakin University, Geelong, Victoria, Australia; ^2^ Cancer Services, Barwon Health, Geelong, Victoria, Australia; ^3^ Department of Pediatrics, The University of Melbourne, Parkville, Victoria, Australia; ^4^ Monash Bioinformatics Platform, Monash University, Clayton, Victoria, Australia; ^5^ Department of Neurosurgery, The Royal Melbourne Hospital, Parkville, Victoria, Australia; ^6^ Department of Surgery, The Royal Melbourne Hospital, The University of Melbourne, Parkville, Victoria, Australia; ^7^ Center for Cancer Research, Hudson Institute of Medical Research, Clayton, Victoria, Australia; ^8^ Department of Molecular and Translational Science, Monash University, Clayton, Victoria, Australia; ^9^ NHMRC Clinical Trials Center, University of Sydney, Sydney, New South Wales, Australia; ^10^ The Jackson Laboratory for Genomic Medicine, Farmington, Connecticut, USA; ^11^ Department of Medical Oncology, Peter MacCallum Cancer Center, Melbourne, Victoria, Australia

**Keywords:** glioblastoma, mutation profiling, neutral evolution, selection pressure, tumor heterogeneity

## Abstract

Glioblastoma presents as a heterogeneous disease with poor prognosis despite the use of multimodal therapy. Analysis of genomic DNA changes between initial diagnosis and recurrence in response to standard treatment protocols would enhance understanding of disease progression and better inform new treatment strategies. A cohort of 21 patients with primary glioblastoma were examined between diagnosis and first recurrence. This study presented a rare opportunity to characterize molecular alterations in tumors observed in three patients who received no therapeutic intervention, other than surgery, offering a unique control. We focused this study by comparing the dynamic mutation profiles between the primary tumors and their matched recurrent counterparts. Molecular profiling of tumors was performed using multiplexed targeted deep sequencing of 409 well characterized cancer-associated genes, achieving a mean read depth of 1272 x. Three levels of evidence suggested an evolutionary pattern consistent with a response to therapy-mediated selection pressures exists in treated patients: 1) variant burden was reduced in recurrent tumors, 2) neutral evolutionary dynamics apparent in untreated tumors shifted toward a non-neutral mode of evolution in treated patients at recurrence, and 3) the recurrent tumor of one patient displayed an increased mutation rate attributable to a temozolomide-associated hypermutator phenotype. Our observations suggest that current treatment modalities are likely to fail in achieving long term remission with the majority of relapse samples containing distinct mutations when compared to primary diagnostic samples.

## INTRODUCTION

Fundamental knowledge of the biology of recurrent glioblastoma (GBM) has been limited until recently. This has been due to the comparatively low numbers of patients (20–30%) undergoing surgery at recurrence due to limited evidence of clinical benefit [1, 2], thus limiting opportunities for molecular characterization of the processes associated with disease resistance and recurrence.

In the setting of low grade glioma, hypermutation induced by temozolomide (TMZ) treatment was observed in a subset of astrocytomas at recurrence [3]. Temozolomide-associated mutations were identified that target the retinoblastoma tumor suppressor (RB) pathway, activate AKT-mTOR signaling and promote tumor growth and metastasis. Interestingly, microdissection revealed that these mutations were confined to regions with mTORC1 activation and high Ki-67 index, suggesting that mutations induced by TMZ conferred a growth advantage. Whether these observations are transferable to primary GBM remains to be explored.

Using data from The Cancer Genome Atlas (TCGA) and 21 paired primary and recurrent GBM, Kim et al. [4] inferred two models for clonal evolution at recurrence. In the ancestral cell origin model, dominant clones in the primary tumor disappear in response to therapy. New mutations are then acquired in refractory ancestral cells causing cell proliferation and tumor recurrence. In the clonal evolution model, major primary disease clones survive treatment and continue to grow. Critically, it was found that key alterations in GBM driver genes frequently present in primary tumors were unlikely to be initiating mutations as they were often absent in the recurrent tumor, mutated at a different base or deleted/amplified with different copy number breakpoints suggesting “intratumoral evolutionary pressures resulting in convergent evolutionary events”. This emphasizes the importance of detailing the characteristics of disease relapse in order to better understand the true ancestral alterations driving GBM development and identify potential therapeutic targets.

Wang et al. [5] analyzed longitudinal genomic and transcriptomic data from 114 patients, including a mix of primary and secondary GBM initial and recurrent tumor pairs. A highly branched evolutionary pattern was observed in which 63% of patients exhibited different expression-based subtypes at diagnosis and relapse. Hypermutation of highly expressed genes was detected in 15% of tumors at relapse, with a clear mutational signature.

Neutral tumor evolution describes the distribution of variant allele frequencies (VAFs) within a tumor and predicts that, although individual subclones possess unique mutational patterns, they expand at similar rates and accumulate linearly with the inverse of their frequency, hence following a 1/*f* power-law distribution [6]. Clonal selection appears to have occurred as an early event prior to tumor growth and the acquisition of numerous passenger mutations results in intratumoral heterogeneity. The contrasting notion is that expansion of subclones is influenced by strong selection pressures to adapt to the tumor microenvironment or in response to treatment modalities. The concept of neutral tumor evolution has not been examined in detail in GBM.

In the current study we have explored a well-defined group of clinically annotated primary GBM tumor samples to examine the natural history of mutational alterations within tumors during progression and in response to treatment. Our results emphasize the inherent heterogeneity of primary GBM at a genomic level and show that GBM is an evolving tumor with or without therapeutic intervention. We explored the concept of “effectively-neutral” evolutionary dynamics in the context of untreated and treated primary GBM, showing a shift toward non-neutral evolutionary expansion of subclonal variants after treatment with radiochemotherapy.

## RESULTS

### Sample characteristics

Our study focused on a group of 21 patients (cohort 1) diagnosed with primary GBM, including two glioblastoma with primitive neuronal component (GBM-PNC) histology at diagnosis. Within this cohort, three patients had elected not to receive any intervention other than surgery and the remaining 18 patients received standard concomitant RT and TMZ [7]. Twelve of the treated patients then received four to six cycles of adjuvant TMZ and three received varied adjuvant therapy as described in Table 1. A variety of treatments, including carmustine, carboplatin and bevacizumab were administered as salvage therapy to some patients after the second surgical resection.

**Table 1:** Summary of patient clinical data

Patient	Gender	Age at diagnosis (years)	Primary diagnosis	Diagnosis of recurrence	Treatment prior to second surgery	Time to recurrence (months)	Survival (months)
1	F	50	GBM	GBM	none	19	36.4
2	F	44	GBM	GBM	Concomitant RT/TMZ only	3	22.0
3	M	49	GBM	GBM	Concomitant RT/TMZ and 4 cycles adjuvant TMZ	8	9.9^a^
4	M	35	GBM-PNC	GBM	Concomitant RT/TMZ and standard adjuvant TMZ	11	12.2
7	F	62	GBM	GBM	Concomitant RT/TMZ and standard adjuvant TMZ	7	11.9
8	M	33	GBM	GBM	Concomitant RT/TMZ and standard adjuvant TMZ	9	18.6
9	M	51	GBM	GBM	Concomitant RT/TMZ and adjuvant AVAglio (+/−BVZ)	9	16.4
10	M	57	GBM	GBM	Concomitant RT/TMZ and 2 cycles adjuvant AVAglio (+/−BVZ)	5	17.1
11	M	74	GBM	GBM	Concomitant RT/TMZ only	3	7.2
12	M	66	GBM	GBM	Concomitant RT/TMZ and standard adjuvant TMZ	11	15.9
14	M	56	GBM	GBM	Concomitant RT/TMZ and standard adjuvant TMZ	14	17.6
15	F	33	GBM	GBM	Concomitant RT/TMZ only	8	14.6
16	M	39	GBM	GBM	Concomitant RT/TMZ and 4 cycles adjuvant TMZ	9	14.5
18	F	63	GBM	GBM	Concomitant RT/TMZ and standard adjuvant TMZ	7	14.3
19	M	67	GBM	GS	Concomitant RT/TMZ and standard adjuvant TMZ, then 2nd line BVZ 8 months	18	19.5
20	F	57	GBM	GBM	Concomitant RT/TMZ and 9 cycles adjuvant AVAglio (+/−BVZ)	15	19.3
21	F	48	GBM-PNC	GBM-PNC	Concomitant RT/TMZ and standard adjuvant TMZ	24	29.2
22	M	43	GBM	GBM	Concomitant RT/TMZ and 4 cycles adjuvant TMZ	10	15.1
26	M	66	GBM	GBM	Concomitant RT/TMZ and standard adjuvant TMZ	8	30.6
28	M	54	GBM	GBM	none	2	13.2
29	M	34	GBM	GBM	none	3	13.8

GBM Glioblastoma; GBM-PNC Glioblastoma with primitive neuronal component; GS Gliosarcoma; BVZ bevacizumab;

AVAglio Phase 3 trial of BVZ plus TMZ and RT in newly diagnosed GBM

^a^Censored

### Mutational and key pathway analysis

**Figure 1:**
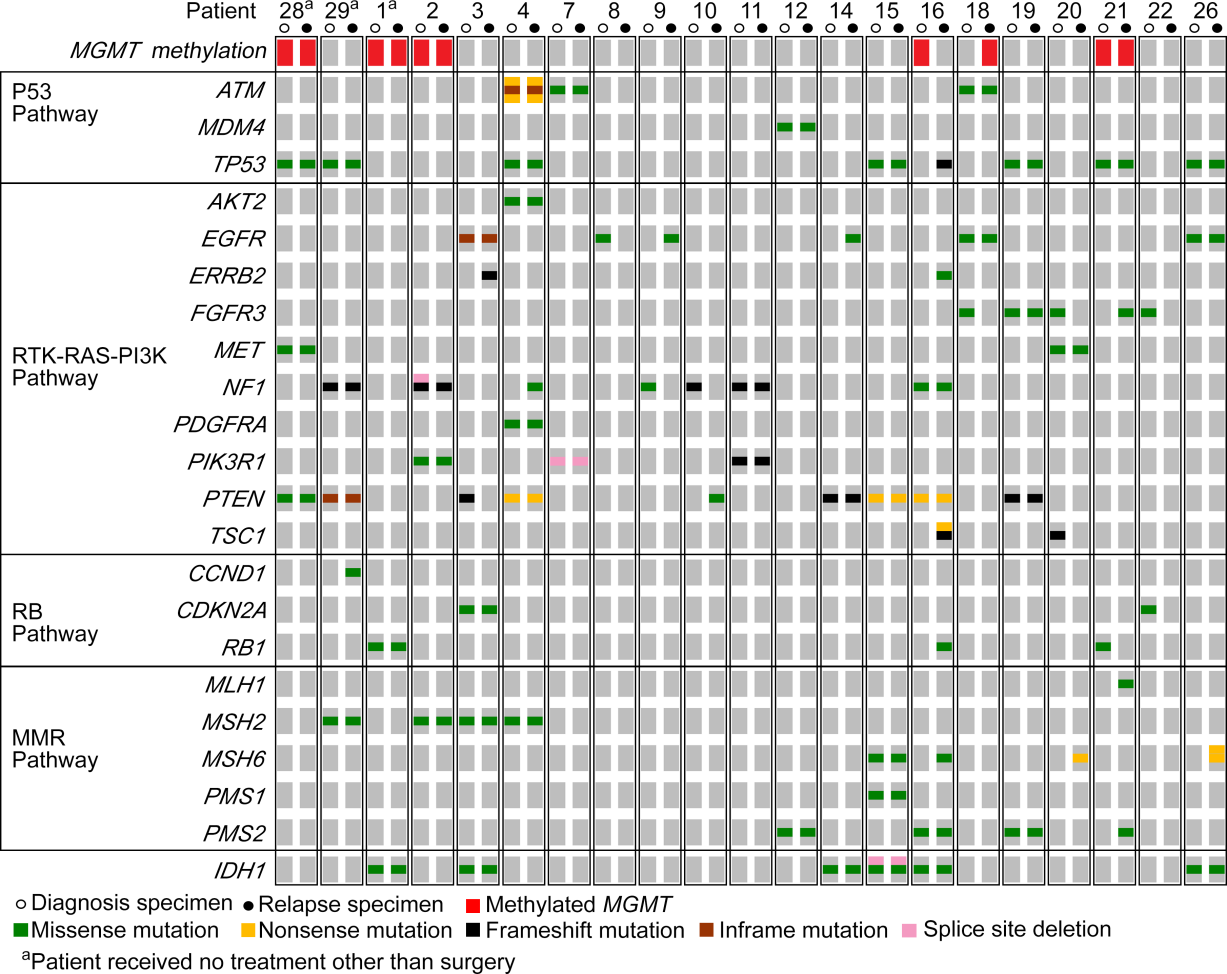
Patterns of mutations in key signaling pathway genes were consistent with previously described traits of primary GBM OncoPrint summarizing the non-synonymous and splice site variants detected by targeted deep sequencing in both diagnostic (open circles) and relapse (closed circles) specimens for each of the 21 patients. Specimens with methylation of MGMT are noted in red.

Specimens from cohort 1 exhibited a pattern of variants in coding regions previously well described for primary GBM [8, 9]. As matched normal specimens were unavailable for this cohort, variant calls may include low frequency and rare single nucleotide polymorphisms (SNPs). The signaling pathways with nonsynonymous or splice site variants detected in key genes are summarized in Figure 1, along with *MGMT* methylation status. Specifically, in their initial tumors, non-synonymous and splice site variants in *EGFR* occurred in four patients (19%) and eight patients (38%) harbored *PTEN* variants, 38% of which were single nucleotide variations (SNVs) resulting in premature stop codons and protein truncation (p.R130*, p.R223*, p.R335*). The overall incidence of *TP53* variants was 33%, detected in seven patients who each possessed distinct SNVs. These were all found to be annotated in the Catalogue of Somatic Mutations in Cancer (COSMIC) [10].

Of significant interest were variants in the mismatch repair (MMR) pathway that occurred in 52% of patients in one or both samples. We observed that in the patients who received RT and TMZ, variants present in *MSH2*, *MSH6*, *PMS1* or *PSM2* at diagnosis were also present at recurrence (Figure 1). Similarly, one of three untreated patients had a variant in *MSH2* that was also present at recurrence. There were no MMR pathway variants unique to diagnosis in any patient and no variants unique to recurrence in the untreated patients. In contrast, in treated patients a number of private variants appeared at recurrence in *MLH1*, *MSH6* and *PSM2,* generally at a frequency of less than 30% suggesting the emergence of subclones in this pathway undetected at diagnosis. Although there was no significant difference in the number of variants detected in key MMR genes in treated and untreated patients overall, the sample in which the variants occurred was noteworthy and was a factor that approached statistical significance (*F*_2, 59_ = 3.13, *p* = 0.051). More specifically, the absence of MMR variants unique to diagnosis, compared to the numbers present in both samples or uniquely at relapse, fell just beyond statistically significant parameters using post-hoc tests (*t*(20) = −2.29, *p* = 0.065 and *t*(20) = 2.03, *p* = 0.113 for shared and unique to relapse comparison respectively; Supplementary Figure 1A).

In the case of *IDH1* we identified six patients with variants common to both their diagnosis and relapse samples, none of which were the well characterized activating substitution at arginine 132 (*IDH1*R132H+) seen in lower grade tumors. Given that somatic *IDH1* variants exclusively alter R132 in GBM as presented in TCGA, these detected variants are likely low frequency or rare SNPs. In this cohort we identified a missense c.548 A > G SNV in Patient 1 (Untreated) and Patient 3 (Treated), resulting in a p.Y183C amino acid change predicted as functionally damaging or deleterious using Sorting Tolerant from Intolerant (SIFT) [11] or Protein Variation Effect Analyzer (PROVEAN) [12] scores respectively. This variant is recorded in the NCBI SNP database (dbSNP rs34599179) as a rare SNP with a minor allele frequency (MAF) of 0.36%. Patients 14, 15, 16, 26 (All Treated) each had a c.532 G > A (p.V178I) missense variant predicted to be tolerated/neutral. This is classified as a low frequency SNP (rs34218846; MAF=4.93%) and is present in COSMIC (COSM97131), confirmed as somatic for thyroid and lymphoid studies [13]. To date a functional role remains unclear. There was also a deletion in the 5′ splice site of exon 9 (NG_023319.2:g.28927delC) in Patient 15, a rare SNP (rs533101765) with a MAF of 0.08%.

Methylation of *MGMT* was detected in the primary tumors of five patients and the methylation persisted at recurrence in four of these patients. An additional patient was *MGMT* methylated uniquely at recurrence (Figure 1). Only one recurrent patient sample (patient 21) showed a pattern consistent with the MMR-defective hypermutator phenotype [14–17]. Similar to previous reports, *MGMT* was methylated and multiple DNA MMR genes (*MLH1* and *PMS2)* were mutated in the recurrent tumor, but these variants were not present at diagnosis. Variants in *IDH1* or *IDH2* were not detected in this patient (Figure 1).

### In primary GBMs standard radiochemotherapy did not impact the burden of mutations in recurrent tumors with the exception of one patient

**Figure 2:**
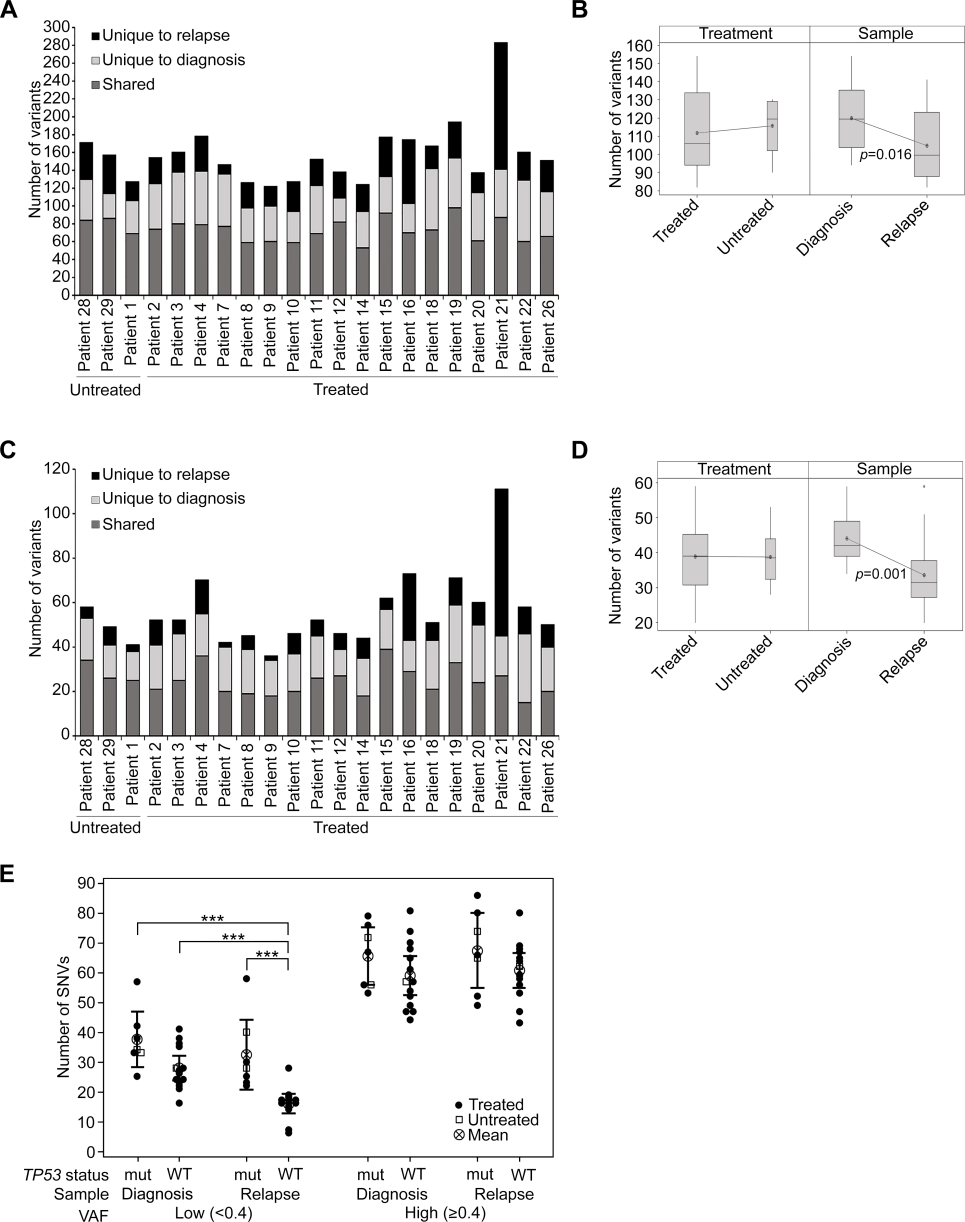
The burden of mutations was not heavily impacted by treatment with RT and TMZ (**A**) The number of variants detected overall in untreated and treated patients, presented according to the specimen/s in which they were observed. (**B**) Box plot showing the mean number of variants by treatment or sample type. Patient 21 was excluded as an outlier from this analysis. (**C**) Coding region variants defined as non-synonymous and splice site mutations, presented as shared or unique to either diagnosis or relapse specimens. (**D**) Box plot showing the mean number of non-synonymous and splice site variants by treatment (treated vs untreated) or sample type (diagnosis vs relapse). Patient 21 was excluded from this analysis. (**E**) The presence of SNVs was analyzed according to the *TP53* status of tumor specimens. *TP53* wildtype tumors (WT) were compared to *TP53* mutant tumors (mut), which were defined as those that contained non-synonymous *TP53* variants. SNVs were designated low VAF (< 0.4) or high VAF (≥ 0.4) and were tallied separately for the diagnosis and relapse specimens of each patient (excluding the relapse sample of patient 21). Untreated patients are depicted as open squares and treated patients as closed circles. The mean and 95% confidence intervals are also shown. A two-way analysis of variance was performed using a general linear model and *p*-values for significant differences (*p* ≤ 0.001) are displayed.

When considering the total burden of genetic variation detected, including intronic, splice site, non-synonymous and synonymous exonic variants (patient 21 was excluded as an outlier; *p* ≤ 0.001), in treated patients there was an average of 120 variants detected in newly diagnosed and 103 variants in recurrent samples. We found an average of 49 variants to be unique to diagnosis and 32 to be unique to recurrence. In the three untreated patients a similar pattern of mutational burden was observed with an average of 117 and 115 variants detected in diagnosis and recurrence samples respectively. An average of 37 variants were unique to diagnosis and 35 were unique to recurrence (Figure 2A). In contrast, patient 21 was found to have 54 variants unique to diagnosis and 142 unique to the recurrent tumor (Figure 2A). Excluding patient 21, the mean number of variants was significantly higher in diagnosis samples compared to relapse (*F*_1,37_ = 6.44, *p* = 0.016) and there was no significant difference between the treated and untreated patients (*F*_1,37_ = 0.22, *p* = 0.641; Figure 2B). The number of variants observed also varied by sample type (*F*_2,56_ = 52.87, *p* < 0.001), with a significantly higher number of variants found to be shared between matched samples than unique to either sample (*t*(19) = 6.36, *p* < 0.001 and *t*(19) = 10.18, *p* < 0.001 for unique to diagnosis and unique to relapse comparison respectively), which may in part be due to the presence of low frequency or rare SNPs. Moreover, the mean number of variants unique to relapse was also significantly lower than those unique to diagnosis (*t*(19) = 3.82, *p* = 0.001). There was no observable effect of treatment on this pattern of variants (Supplementary Figure 1B, 1C; Supplementary Table 1).

Considering only coding region variants that were non-synonymous and splice site variants and therefore of potentially more functional significance (Figure 2C; Supplementary Table 1; Supplementary Table 2), once again no difference in variant burden was observed between treated and untreated patients (*F*_1,37_ = 0.00, *p* = 0.996), again with the exclusion of patient 21. Significantly fewer variants were detected at recurrence (*F*_1,37_ = 14.28, *p* = 0.001; Figure 2D). The mean number of variants unique to relapse remained lower than those unique to diagnosis (*t*(19) = 5.64, *p* < 0.001) and again there was no observable effect of treatment (Supplementary Figure 1D, 1E).

In line with recent data [4], the presence of non-synonymous *TP53* variants was associated with a higher incidence of low frequency (VAF < 0.4) SNVs in recurrent tumors (*F*_1,38_ = 21.73, *p* < 0.001; Figure 2E). Tumors were designated *TP53* mutated if they possessed a non-synonymous variant that was predicted to be damaging/deleterious by SIFT/PROVEAN. All of these variants were also previously confirmed as somatic in other glioma tumor samples presented in COSMIC. There was no significant difference between *TP53* wildtype and mutant tumors at diagnosis, however, the disproportionate variant numbers overall between primary and recurrent specimens was not observed in *TP53* mutated tumors and was confined to low frequency variants in *TP53* wildtype tumors (Figure 2E; Supplementary Figure 1F). These effects were not evident for high frequency variants (VAF ≥ 0.4) (Figure 2E; Supplementary Figure 1G).

### Treatment with TMZ was found to have little effect on the generation of sequence variation in recurrent GBM

**Figure 3:**
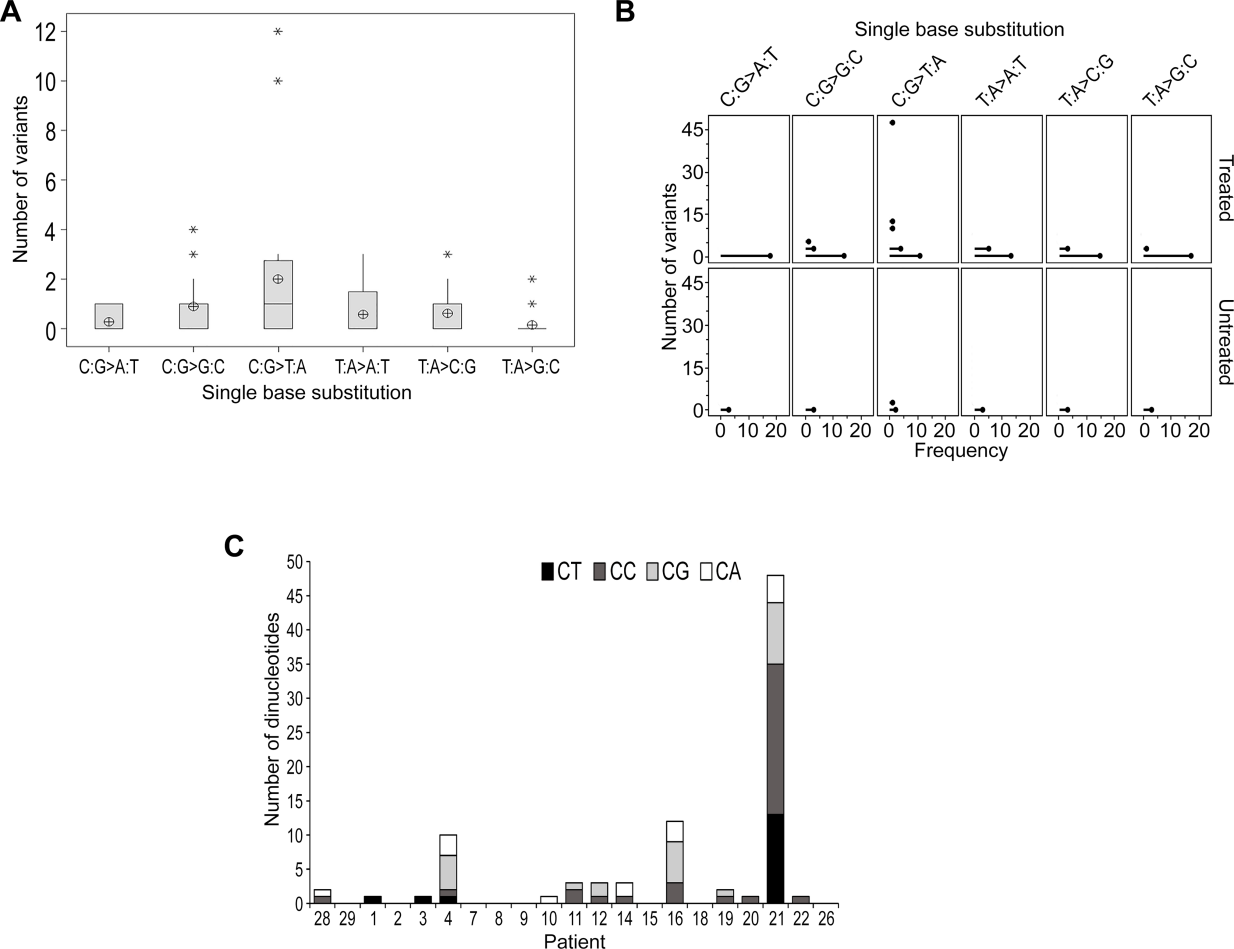
TMZ-induced mutational events were not evident in the majority of samples at recurrence (**A**) Boxplot representing the distribution of single base substitutions unique to recurrence. The y-axis excludes patient 21 as an outlier with 48 detected variants. The mean is shown as a circular symbol and the asterisks highlight outliers. (**B**) An individual value plot presenting the spread of single base substitutions occurring uniquely at recurrence and the frequency of patients in which they occur. Substitutions detected in treated patients (including patient 21) and untreated patients are highlighted. (**C**) Total number of C:G > T:A substitutions and the proportion of each dinucleotide context.

A recognized signature of TMZ-induced mutagenesis is the production of C > T/G > A transitions, predominantly occurring at CpC and CpT dinucleotides. Analysis of the distribution of single base substitutions unique to recurrence across all patients revealed a higher number of C > T/G > A base transitions overall (*F*_1,113_ = 3.98, *p* = 0.002; Figure 3A). However, no clear difference was identified when comparing untreated to treated patients after adjusting for the type of single base substitution (*F*_1,113_ = 1.85, *p* = 0.176; Figure 3B).

We observed TMZ treatment-induced hypermutation, previously described in secondary GBM [3, 18], in the recurrent tumor of only patient 21. Approximately 72% of the SNVs identified at recurrence were classified as TMZ-associated C:G > T:A variants unique to relapse and 73% of these occurred at CpC and CpT dinucleotides, characteristic of TMZ-induced hypermutation (Supplementary Table 3). Review of the pathology status did suggest this patient was histologically distinct as a GBM-PNC and the patient survived for an extended period of 29 months.

In these analyses we compared the total number and proportions of each dinucleotide context of these variants (Figure 3C; Supplementary Table 3) and, excluding patient 21, we could not discern any enrichment for these TMZ-associated transitions in any patient.

### Neutral tumor evolution was prominent in untreated GBM and a shift toward non-neutral evolutionary dynamics was evident in subclones after treatment

**Figure 4:**
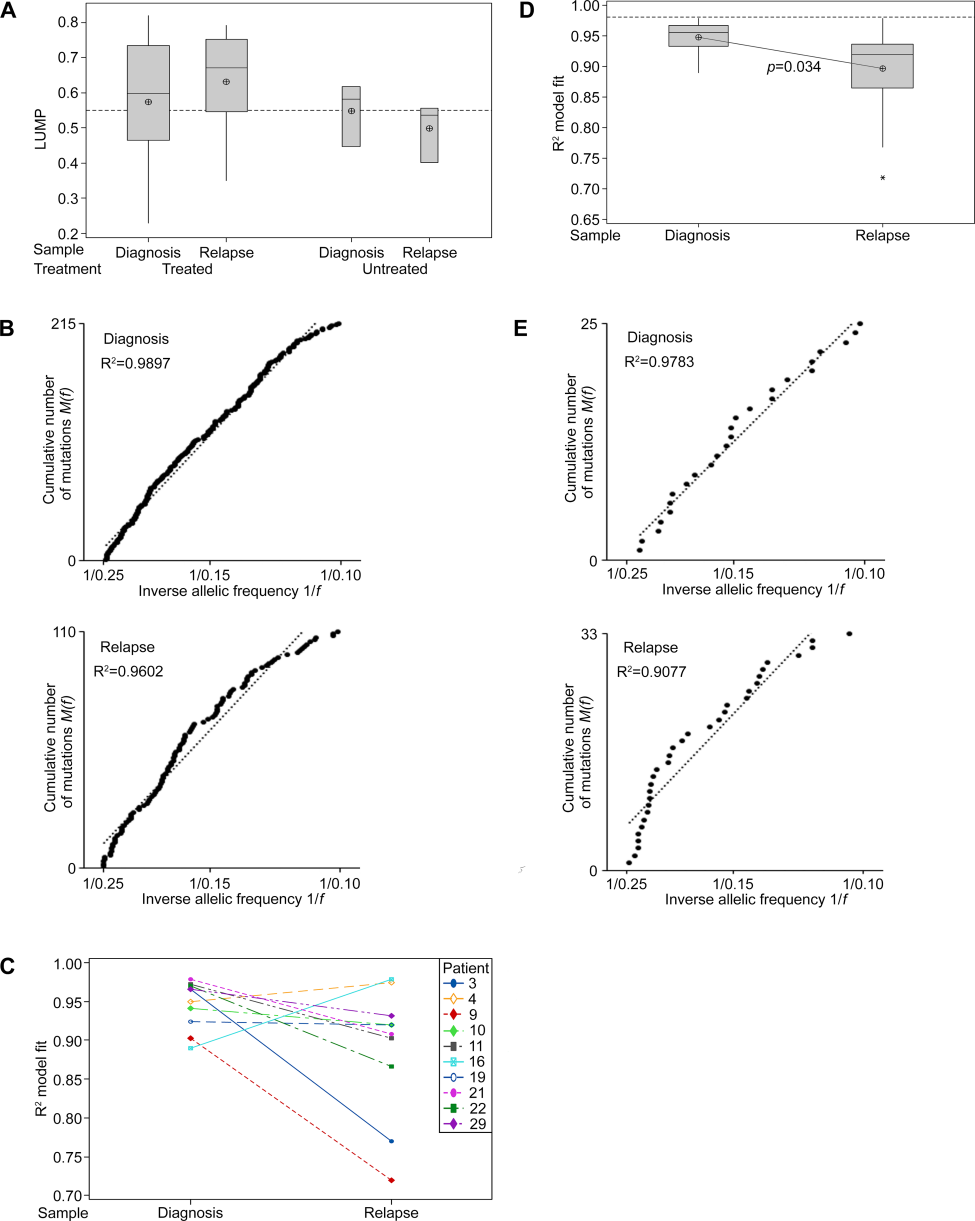
Neutral tumor evolution was evident in untreated GBM, however, a shift away from effectively-neutral subclone dynamics was observed after radiochemotherapy (**A**) Boxplot presenting the estimated purity of samples determined by LUMP analysis. The line of short dashes indicates the 55% purity threshold for inclusion in neutral evolution analyses. (**B**) The cumulative distribution *M(f)* of subclonal mutations in primary tumors prior to therapy analyzed collectively was linear with the inverse of their frequency (1/*f*) (upper panel) and a shift away from linearity and therefore neutral evolution was evident in tumors after treatment at recurrence (lower panel; excludes patient 21 recurrent sample). A stringent *R*^2^ > 0.98 defines neutral evolution in these collective analyses. (**C**) Subclonal mutations of individual samples were assessed and the variation between tumors at diagnosis and relapse are presented for the 10 paired samples analyzed. *R*^2^ goodness-of-fit measures for the neutral evolution model decrease from diagnosis to recurrence in 80% of paired samples from the same patient. (**D**) Boxplot of the *R*^2^ goodness-of-fit for the neutral evolution model. Subclonal mutations of individual samples were assessed and the variation between tumors at diagnosis and relapse are presented for all samples analyzed. The line of short dashes indicates the *R*^2^ = 0.98 threshold used to distinguish neutral and non-neutral evolution. A two-way analysis of variance was performed using the general linear model for continuous variables and the corresponding *p*-value is displayed. (**E**) The cumulative distribution *M(f)* of subclonal mutations in the primary tumor (upper panel) and relapse tumor (lower panel) of patient 21. A switch from neutral to non-neutral evolutionary dynamics was apparent between tumors at diagnosis and relapse.

Leukocytes unmethylation to infer tumor purity (LUMP) analysis, as detailed previously [19], was undertaken for 20 of the matched samples in cohort 1 (patient 12 was excluded due to sample quality limitations). A sample purity threshold of 55% excluded seven diagnosis and six recurrent samples, which included three of six specimens from untreated patients (Figure 4A; Supplementary Table 4). To account for the potential confounding effects of purity, the VAFs detected in the remaining specimens were corrected to adjust for varying levels of normal tissue contamination [6].

Neutral evolution was evident in the tumors of patients at diagnosis when subclonal variants and their frequency were analyzed collectively. As predicted by the neutral evolution model, the cumulative distribution of subclonal variants was found to be linear with the inverse of their frequency. A strong fit to the neutral evolution model with an *R*^2^ value of 0.9897 (Figure 4B) was observed overall for primary tumors and a shift to non-neutral evolutionary dynamics was seen at recurrence (*R*^2^ = 0.9571), suggesting that the selective pressures of radiochemotherapy influenced subclonal variant expansion. Excluding the relapse sample of patient 21 from this analysis, due to its TMZ-associated hypermutated status, resulted in a slight increase in the goodness-of-fit for the neutral evolution model at recurrence (*R*^2^ = 0.9602; Figure 4B). A non-neutral evolutionary dynamic persisted suggesting that selection pressures at recurrence in treated patients were not unique to the MMR-defective hypermutator phenotype.

Due to the targeted nature of this high-depth sequencing analysis, goodness-of-fit measures using the stringent threshold of *R*^2^ > 0.98 for defining neutral evolution in individual specimens may undercall neutrality as low numbers of variants with which to fit the model may lead to a poorer fit to the data [6]. In addition to discriminating between neutral and non-neutral evolution in primary tumors, we were able to compare the dynamics of subclonal variants between diagnosis and first recurrence, reflecting treatment influences. Determination of individual goodness-of-fit measures for each sample showed that in paired diagnosis and relapse specimens from the same patient the neutral evolution model fit decreased in 8 of 10 patients (Figure 4C). Subclonal variants in 5 of 13 primary tumor specimens achieved *R*^2^ values above 0.96. The mean *R*^2^ value of samples at diagnosis was not significantly different from an *R*^2^ value of 0.96 (*M* = 0.9477, 95% CI [0.9314, 0.9640], *t*(12) = −1.65, *p* = 0.125). Whilst collectively primary samples displayed effectively-neutral evolution, analysis of specimens individually failed to designate any samples as evolving neutrally. The mean *R*^2^ value obtained for patients at diagnosis was significantly lower than the stringent *R*^2^ threshold of 0.98 (*t*(12) = −4.832, *p* = 0.001). In contrast to diagnostic samples, the mean *R*^2^ value of relapse samples was significantly lower than an *R*^2^ value of 0.96 (*M* = 0.89654, 95% CI [0.8494, 0.9414], *t*(12) = −3.06, *p* = 0.01). At recurrence, all 13 samples were classified as undergoing non-neutral evolutionary expansion of subclonal variants. A further shift away from a neutral model fit was evident in the majority of samples at recurrence with significantly higher *R*^2^ values observed for diagnosis compared to relapse specimens (*F*_1,24_ = 5.06, *p* = 0.034; Figure 4D). Excluding the recurrent sample of patient 21 does not substantially alter the significance of the difference observed between samples at diagnosis and relapse (*F*_1,23_ = 4.89, *p* = 0.037). Neutral evolution of subclonal variants was evident in the primary tumor of Patient 21 (*R*^2^ > 0.9783). However, in the recurrent tumor with TMZ-associated hypermutation non-neutral dynamics dominate (*R*^2^ > 0.9077; Figure 4E). These observations suggest that radiochemotherapy exerts selective pressure influencing subclonal variant dynamics in treated tumors.

In an attempt to verify these observations in a larger cohort, publicly available tumor datasets were interrogated for purity and VAF information. Two sets of data were analyzed which included selected samples (cohort 2) from TCGA [20] and additional samples (cohort 3) previously collated and reported by Wang et al. [5]. A total of 408 samples from TCGA were considered, including 12 recurrent specimens. Of these, 175 were evaluated for neutral tumor evolution with data from 93 primary samples satisfying final inclusion criteria for analysis (Supplementary Table 4). Criteria for exclusion were absent or low purity estimations as determined by the ABSOLUTE computational method [21] and insufficient numbers of subclonal variants identified for analysis. Cohort 3 consisted of 10 specimens, from six primary and four recurrent tumors. A total of 142 samples were excluded for the above-mentioned reasons. The remaining 10 samples were all primary GBM with no history of lower grade glioma, nine of which were from the INCB cohort from the Besta Brain Biobank, previously published by Wang et al. [5]. The final sample was from the SMC cohort from Seoul National University Hospital, previously reported by Kim et al. [22]. This exercise highlighted the challenges of analyzing disparate datasets with absent parameters or collected using varied methodology. Approaches varied for both sequencing, including platform and depth of coverage, and inference of tumor purity. The three cohorts were therefore not directly comparable, limiting observations to three smaller individual cohorts. The higher rates of exclusion to obtain cohorts 2 and 3 may also be reflective of broader GBM subtype representation within the cohorts, and the potential purity differences between them [19].

**Figure 5:**
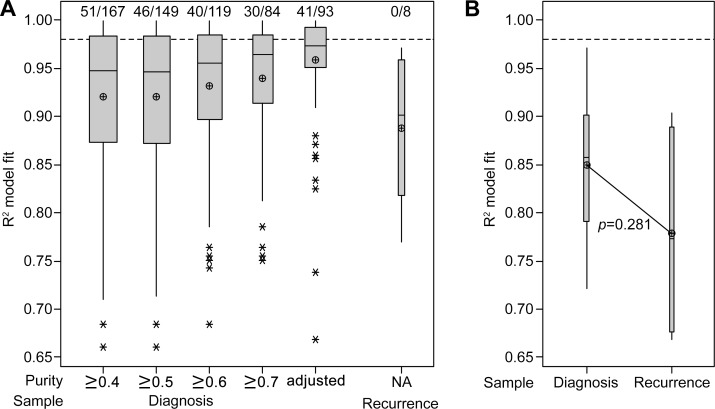
Interrogation of publically available datasets also indicates a stronger fit to the neutral evolution model in untreated primary tumors (**A**) Boxplot of the *R*^2^ goodness-of-fit for the neutral evolution model, presenting data from cohort 2. The minimum purity level of included samples is categorized below the x-axis and the 93 samples which satisfied inclusion criteria for analysis using purity-adjusted VAFs are compared. The line of short dashes indicates the *R*^2^ = 0.98 threshold used to distinguish neutral and non-neutral evolution and the number of samples greater than this threshold out of the total number analyzed in each category is indicated above the dashed line. (**B**) Boxplot of the *R*^2^ goodness-of-fit for the neutral evolution model, presenting data from cohort 3. The line of short dashes indicates the *R*^2^ = 0.98 threshold. A two-way analysis of variance was performed using the general linear model for continuous variables and the corresponding *p*-value is displayed.

The effects of including samples to a range of minimum purity levels was analyzed in the larger group (cohort 2) of primary samples from TCGA (Figure 5A). Whilst lowering the acceptable minimum purity value increased the number of samples included in the analysis, it resulted in poorer neutral evolution model fits. In comparison, use of purity-adjusted VAFs revealed more similar model fits (*Mdn* = 0.9738, 95% CI [0.9647, 0.9824]), with 44% of samples dominated by neutral evolutionary dynamics as defined by an *R*^2^ value of at least 0.98. There seemed to be a shift away from characteristically neutral evolution in recurrent samples (*Mdn* = 0.9011, 95% CI [0.8098, 0.9606]). However, the analysis was limited to only eight samples with no available purity data, and therefore offers little insight. Similarly, cohort 3 consisted of 10 specimens with purity data enabling adjustment of VAFs prior to assessment of a neutral evolution model fit. The *R*^2^ model fit values at recurrence again were seemingly lower than at diagnosis (*Mdn* = 0.8574, 95% CI [0.7542, 0.9382] at diagnosis vs *Mdn* = 0.7728, 95% CI [0.6685, 0.9032] at recurrence), but this difference did not reach statistical significance (*p* = 0.281; Figure 5B).

Together, these data suggest that there is a stronger fit to a neutral evolution model in GBM samples at diagnosis, shifting quickly to dynamics reflecting selection pressures at recurrence.

## DISCUSSION

Our study is unique and adds several new findings to the field. We were able to interrogate untreated (*n* = 3) and treated (*n* = 18) patients and confirmed the dynamic nature of genomic alterations in GBM. The instability of genomic alterations after GBM recurrence relative to GBM primary tumor samples was evident, even in the absence of any therapy. In contrast to data published by others in primary GBM [4, 5] and previously described in secondary GBM [3], we only observed TMZ treatment-induced hypermutation in the recurrent tumor of one patient. Consistent with previous reports, the majority of variants were of low VAF and occurred in multiple DNA MMR genes. *MGMT* was also methylated in both the primary tumor and at recurrence. In contrast to other studies, both were *IDH* wildtype [3, 22] providing one example against the notion that the lack of hypermutation in primary GBM is due to a fundamental difference between *IDH* mutant (secondary) and *IDH* wildtype (primary) GBM [23]. Furthermore, when closely examining the total number and proportions of TMZ-associated variants in each dinucleotide context, we could not discern enrichment for these TMZ-associated transitions in any patients other than the one patient. This suggests a different evolution of variants or active repair of such variants in the majority of primary GBM after TMZ treatment.

**Figure 6:**
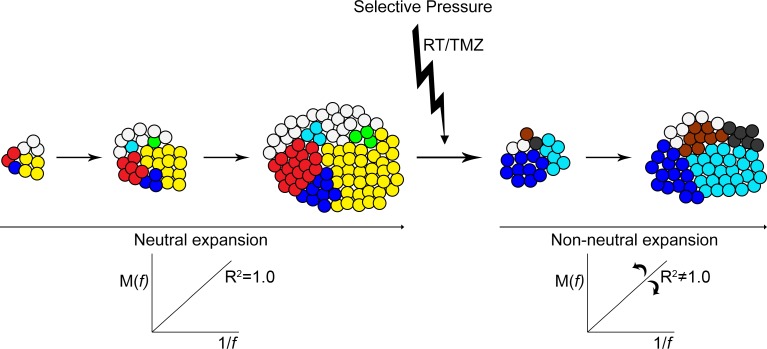
Neutral expansion of variants shifts toward non-neutral modes after therapy Pictorial representation of the neutral expansion of subclones and the linear accumulation of variants *M(f)* with the inverse of their frequency *f*. An *R*^2^ equal to 1.0 depicts precisely neutral evolutionary dynamics. With the applied pressure of therapy subclonal expansion becomes altered and variable. Some resistant clones and clones with treatment-induced mutations experience a growth advantage and expand rapidly, while other treatment-sensitive clones are reduced or eliminated. The allelic frequency of some variants increases at a more rapid rate while others are decreased. Non-neutral modes of subclonal variant expansion dominate.

A perfectly neutral evolution of subclonal variants results in a constant allelic frequency of each variant as the tumor expands (Figure 6). The growth rate of cells that possess one or more variants is no different from cells that do not. New variants that arise expand at the same rate with no fitness advantage. After therapy at recurrence a further shift toward non-neutral modes of evolution was observed. A differential response to therapy and treatment-induced mutagenesis would see some resistant mutant subclones expand rapidly while others are eliminated. The allelic frequencies of variants therefore would also alter to varying degrees.

A limitation of this study was its retrospective nature and the unavailability of normal specimens for germline analysis from these patients. Four measures were taken to overcome this limitation while taking full advantage of these matched initial vs. recurrent tumor sets: (1) common variants according to the NCBI SNP database [24] and 1000 genomes MAF data [25] were excluded to limit analyses to probable disease associated variants that impact function; (2) deep sequencing was performed for only previously identified cancer-associated genes with alterations frequently suggestive of gain- or loss-of-functions; (3) neutral evolution analysis assessed subclonal variants with VAFs of less than 0.25 and therefore the potential confounding effects of ploidy and low MAF germline variants were avoided; (4) analyses were centered around comparing the dynamic mutation profiles between the primary tumors and their matched recurrent counterparts (i.e. we did not aim to identify novel cancer genes). Choice of platform was primarily determined by the limited material available for processing. The neutral evolution model posits that the expanding tumor randomly accumulates a large number of new variants in continually smaller subclonal fractions. Distinct variants are identified in different regions, however, they follow the same *1/f* distribution. Whilst a greater breadth of sequencing would have facilitated a more thorough assessment of variant dynamics, the comprehensive cancer panel provided a snapshot view comparing the changes in evolutionary dynamics in response to treatment rather than a single timepoint. Additionally, a higher read depth provided more accurate detection of subclonal variants and ensured sufficient coverage even in regions with greater sequence complexity. Future prospective studies including germline analysis will be needed.

Whilst the size of our cohort was not sufficient to detect subtle disease pathway associations at the levels of statistical significance we were able to make several significant observations. We did observe an increase in the number of variants with low VAF (< 0.4) in patients with mutated *TP53* at recurrence confirming previous observations [4]. The variant data presented here have a number of implications for patients and future approaches to therapy. Whilst there was a clear molecular response to therapy with DNA damaging agents, the dynamic and rapidly evolving nature of variants in GBM conspires to quickly select clonal populations that are resistant to these therapies. The majority of patients fail treatment quickly and succumb to the disease. The median time to recurrence whilst on treatment of our cohort as an example was only nine months. These data confirm for those in the field that such approaches with treatment modalities using DNA damage are highly unlikely to translate to long term success in treatment. Additionally, the data are also suggestive that single targeted therapies using a one-dimensional personalized approach are likely to be quickly overcome through non-neutral evolutionary selection. An alternative approach using sequential interventions and a chronic disease therapeutic and management paradigm may be one consideration. This study also highlights a need for a concerted effort to advance GBM research, employing prospectively secured specimens and implementing standardized methodologies and data analysis pipelines.

## MATERIALS AND METHODS

### Biospecimen and data acquisition

Formalin-fixed paraffin-embedded (FFPE) tumor samples were obtained from the Royal Melbourne Hospital (RMH) and University of Melbourne, Department of Surgery Brain Tumor Bank. Tissue was collected after informed consent during surgical resection at diagnosis and recurrence, and matched clinical data was obtained from the Australian Comprehensive Cancer Outcomes and Research Database (ACCORD). Specimen use was approved by the Melbourne Health Human Research Ethics Committee (HREC; 2013.084) and research was approved by the Monash Health HREC (1301A), with subsequent site approvals by the Deakin University (2013–124) and Barwon Health HRECs (13/16).

Additional data on GBM samples from publicly available datasets were interrogated for VAF and purity information. The NCI Genomic Data Commons (GDC) [26] portal was used to access the MuTect masked somatic mutation MAF file format of the TCGA-GBM dataset (https://gdc-portal.nci.nih.gov/projects/TCGA-GBM, download November 2016). ABSOLUTE purity levels for the dataset were collated from the cBioPortal for Cancer Genomics merged cohort of LGG and GBM data file (http://www.cbioportal.org/study?id=lgggbm_tcga_pub#clinical, download November 2016) [27]. Variant frequency and purity data was gathered for a further nine samples from the INCB cohort from the Besta Brain Biobank, previously published by Wang et al. [5] and one sample from the SMC cohort from Seoul National University Hospital, originally reported by Kim et al. [22].

### DNA extraction

Genomic DNA was extracted from tumor tissue using the ReliaPrep™ FFPE gDNA Miniprep System (Promega), as directed by the manufacturer. The quality of the DNA was assessed using a nanodrop spectrophotometer and quantified using a Qubit^®^ dsDNA HS Assay kit and a Qubit^®^2.0 Fluorometer (ThermoFisher).

### Targeted sequencing

Multiplexed targeted sequencing was performed using the Ion AmpliSeq™ Comprehensive Cancer Panel which interrogates 409 cancer associated genes, utilizing 16000 primer pairs in four pools. Barcoded libraries were prepared using 10 ng of gDNA per primer pool and the Ion AmpliSeq™ Library Kit 2.0 (Life Technologies), as directed by the manufacturer. Template preparation and sequencing was conducted by the MHTP Medical Genomics Facility (Melbourne, Australia). Amplified libraries were quantified using an Agilent^®^ 2100 Bioanalyzer and combined equally to a final concentration of 100 pM for template preparation with the Ion PI™ Template OT2 200 Kit v3. Libraries were sequenced on an Ion Proton™ system using the Ion PI™ Sequencing 200 kit v3 and four samples (16 libraries) were loaded onto each Ion PI™ chip v2.

### Data processing and variant identification

For cohort 1, sequencing reads were aligned to the Human hg19 reference sequence (Build 37) and variant calling was performed using the Ion Torrent Software Suite 4.0.2 and the Somatic-Proton-Low Stringency Variant Caller Plugin v4.0-r76860. Ion Reporter™ Software v4.6 was used for variant annotation and functional prediction and the Broad Institute Integrative Genomics Viewer (IGV) was used for manual visualization and exclusion of some variants. On average 21 million mapped reads (range 12.4–28.3 million) with a mean read length of 108 bp (range 103–112) were generated and were 99% on-target. A mean read depth of 1272 (range 769–1697 reads) was achieved with an average of 81% uniformity of coverage (range 69–91%). Overall, 89% of bases were called with a predicted quality score of at least 20 (Q ≥ 20), encompassing 2 × 10^9^ bases (range 1.3 × 10^9^−2.7 × 10^9^ bases). Stringent quality, coverage and frequency metrics were applied to detected variants to restrict analyses to high confidence variants and eliminate possible sequencing artefacts [28]. The filtering criteria used selected variant calls with *p*-values of less than 0.05, quality scores of at least 20 (Q20), coverage of greater than 100 reads, allele read counts of at least 50 reads, and VAFs of at least 8%. Common variants with a minor allele frequency, according to the 1000 genomes database, of greater than 5% were also excluded to limit analyses to probable disease associated variants that impact function. Variants that occurred in more than 50% of patients were excluded as likely artefacts. The cBioPortal for Cancer Genomics OncoPrinter was used to generate OncoPrints of key pathway variants [29, 30].

A neutral evolution model was applied to variant data as detailed previously [6]. Briefly, the total SNVs detected within tumor specimens, limited to samples with less than or equal to 45% normal contamination as determined by leukocyte unmethylation to infer tumor purity (LUMP) analysis and variants with purity-adjusted allele frequencies greater than 0.10 and less than 0.25, encompassing subclonal variants reliably detected by high-throughput sequencing, were used to fit the model. Therefore clonal events and the potential confounding effects of ploidy and low MAF germline variants, which would occur at VAFs greater than 0.25, were eliminated. A stringent threshold of *R*^2^ > 0.98 was used for defining the goodness-of-fit to the neutral evolution model. In samples with fewer than 10 variants, results were scrutinized to ensure that a single outlying data point did not heavily influence the slope of the fitted line and alter the detection of neutral or non-neutral evolution. Due to the targeted nature of this high-depth sequencing analysis, fewer subclonal variants available to fit the model may undercall neutrality when individual specimens are analyzed [6]. Assessment of publicly available data was limited to samples with less than or equal to 45% normal contamination as determined by ABSOLUTE purity values. Due to the lower depth of sequencing, only samples with a minimum count of 12 subclonal variants with purity-adjusted allele frequencies greater than or equal to 0.10 or less than 0.25 were considered.

Consideration of SIFT [11] and PROVEAN [12] scores was used for the prediction of the functional impact of exonic variants.

### Variant validation

A semi-selected validation approach was undertaken, focusing on coding variants in genes involved in the key signaling pathways altered in GBM. In order to be reliably detected by Sanger sequencing only variants with an allele frequency greater than 20% were sequenced (primer details Supplementary Table 5). These totaled 46 variants, including 36 SNVs and 10 small insertions and deletions (INDELs) and involved the sequencing of 138 out of the 5357 (2.6%) variants detected overall. Similar to other studies [3, 4, 31], validation rates for SNVs and INDELs were 94% and 50% respectively, achieving an overall variant validation rate of 85%.

### DNA methylation analysis of MGMT

Genomic DNA was prepared for analysis using the MethyEasy™ Bisulphite Kit (Genetic signatures) and subsequent processing was undertaken by The Centre for Applied Genomics, The Hospital for Sick Children (Toronto, Canada) as per manufacturer's instructions. Four samples were replicated on dual array processing runs to assess the reproducibility of the arrays. Data for each replicate sample correlated well (average *R*^2^ = 0.952; range 0.904–0.986). The methylation status of *MGMT* was determined by assessing both the beta value determined for probe cg12981137, which detects the level of methylation at a CpG site within a distinct region of *MGMT* exon 1 found to be most strongly associated with expression [32], and the mean beta value across all 12 available probes corresponding to sites in the promoter region of *MGMT*. Samples with a beta value above a threshold of 0.2 as described previously [33] for probe cg12981137 that also correlated with a mean beta value above this threshold across all 12 sites within the promoter region were considered methylated.

### Leukocytes unmethylation to infer tumor purity analysis

Due to the absence of matched normal DNA, LUMP analysis was used to assess tumor purity of samples in cohort 1 as previously described [19]. LUMP estimations were based on the average methylation levels of 39 of the informative CpG sites. Probes cg00240653, cg02997560, cg03436397, cg26427109 and cg05199874 were excluded from the analysis as the detection *p*-value for these was not below a threshold (*T*) of 0.05.

### Statistics

All statistical analyses were conducted using the Minitab^®^ v17.1.0 software package. A two-way analysis of variance was performed using the general linear model for continuous variables. Initially a test for the interaction of factors was included. If no significant interaction was identified the test was re-run without the interaction terms. The response, the number of variants, was examined against and adjusted for treatment and sample type or whether a variant was shared or unique between matched samples. The *F-*value *F*(*df* effect, *df* error) and *T*-value *t*(*df*) are also reported where appropriate. Similar analyses were undertaken to examine the differences in variant numbers with specific single base substitutions and between neutral evolution *R*^2^ model fit values. Post-hoc pairwise comparisons were conducted using the Tukey method. The Grubbs’ test was used to detect outliers in the data set. The null hypothesis that there were no outliers was rejected at the 0.05 significance level.

The differences between the number of tumors adhering to the neutral evolution model at diagnosis and relapse were also assessed using a one-sample *t*-test against the threshold value of *R*^2^ = 0.98. A similar analysis was undertaken to compare the mean *R*^2^ model fit values of each group to *R*^2^ = 0.96. For all analyses a *p*-value less than or equal to 0.05 was considered to indicate statistical significance.

### Data access

Sequencing data has been submitted to the Sequence Read Archive (SRA) under accession number SRP080827.

## SUPPLEMENTARY MATERIALS FIGURES AND TABLES








