# Supplementary material for: The evolutionary pattern of mutations in glioblastoma reveals therapy-mediated selection
Source: Oncotarget. 2017 Dec 15;9(8):7844–58. doi: 10.18632/oncotarget.23541 (PMC5814263; doi:10.18632/oncotarget.23541)
Supplement: Supplementary file 1 [file oncotarget-09-7844-s001.pdf]

The evolutionary pattern of mutations in glioblastoma reveals therapy-mediated selection

SUPPLEMENTARY MATERIALS

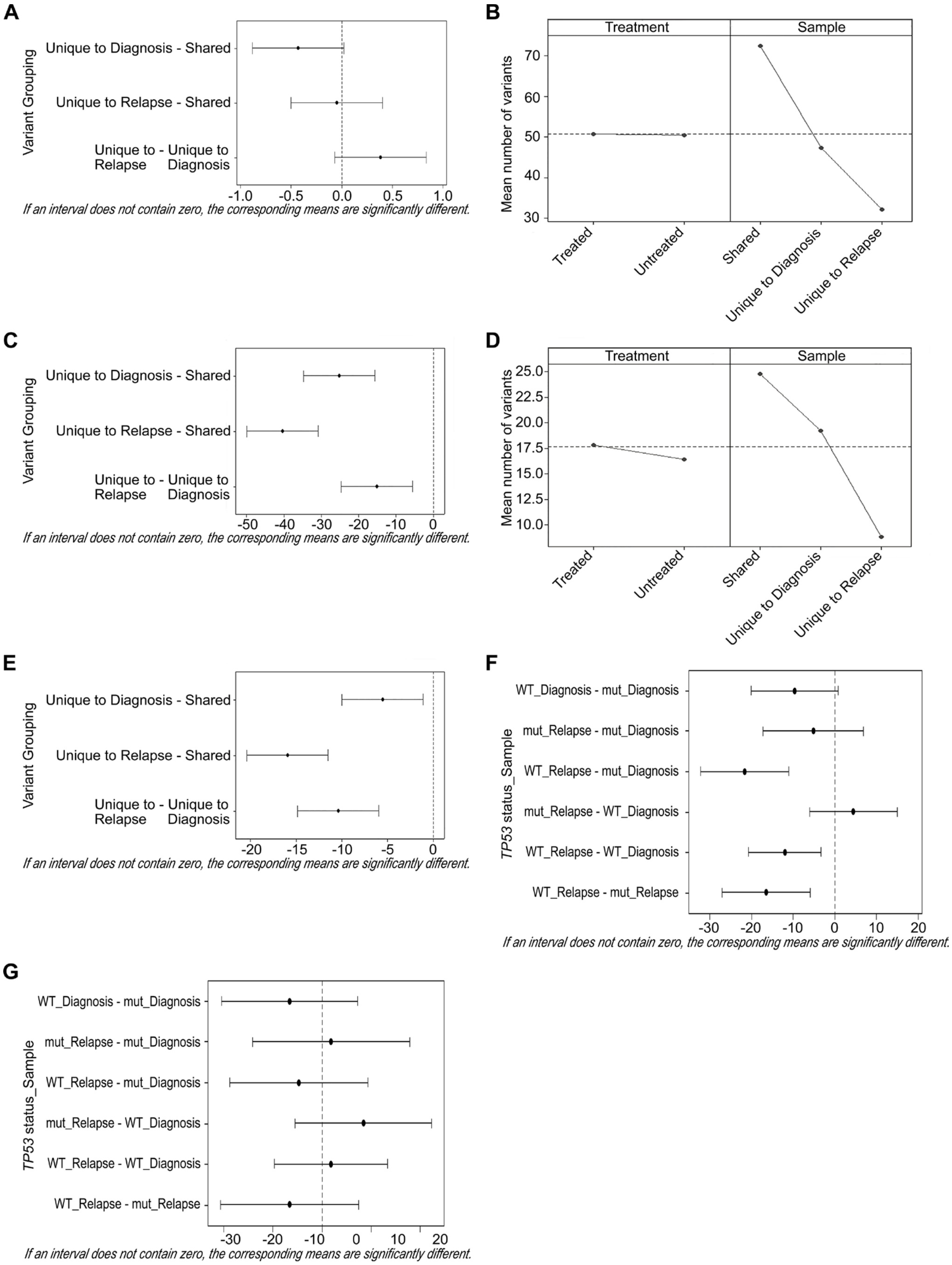

Supplementary Figure 1: (**A**) Tukey significance test of differences observed between the mean number of MMR variants shared or unique to a particular sample. (**B**) Main effects plot depicting the number of all detected variants. (**C**) Tukey significance test of differences observed between the mean number of variants shared or unique to a particular sample. (**D**) Main effects plot depicting non-synonymous and splice site variants and (**E**) the significance of differences observed between means for this subgroup of variants. Tukey significance testing of the effect of TP53 status on differences been the mean number of (**F**) low frequency (< 0.4) and G. high frequency (≥ 0.4) variants. Comparisons between the each possible combination of *TP53* status (wildtype WT; mutant mut) and sample type (diagnosis; relapse) are depicted.

Supplementary Table 1: Summary of the types of variants detected in patient samples. See_Supplementary_Table 1

Supplementary Table 2: Details of non-synonymous and splice site variants. See_Supplementary_Table 2

Supplementary Table 3: Proportion and dinucleotide context of TMZ-associated SNVs unique to recurrence

Patient	Total number of C > T/G > A variants	% C > T/G > A variants	% CpT & CpC	% CpG	% CpA
28	2	11	50	0	50
29	0	0	0	0	0
1	1	8	100	0	0
2	0	0	0	0	0
3	1	6	100	0	0
4	10	27	20	50	30
7	0	0	0	0	0
8	0	0	0	0	0
9	0	0	0	0	0
10	1	7	0	0	100
11	3	18	67	33	0
12	3	23	33	67	0
14	3	23	33	0	67
15	0	0	0	0	0
16	12	39	25	50	25
18	0	0	0	0	0
19	2	10	50	50	0
20	1	7	100	0	0
21	48	72	73	19	8
22	1	10	100	0	0
26	0	0	0	0	0

Supplementary Table 4: Tumor purity estimates and assessment of neutral evolution model fit. See_Supplementary_Table 4

Supplementary Table 5: Primer sequences

	Forward primer	Reverse primer
*EGFR*_ex13	TGCTCTGTCACTGACTGCTG	CCAAAACCTCCAAAAGCCAAGG
EGFR_ex15	TTTGGCTTTCCCCACTCACA	CTGTGGGGACCAAAACACCT
EGFR_ex28	GGACAACCCTGACTACCAGC	TGCAAAACCAGTCTGTGGGT
ERBB2_ex11	GACTGCCTGGTATGTGCCTC	CAAAGCAAGATGCAGACAGTGC
FANCD2_ex9	CTGGGTAATGTGGCTGCAGTTCTA	TGCATACTGCAGTGATAGAACCC
IDH1_ex8	GGTCATTTGGTTGTGGTGGG	CTGCTAAACCTCAGTTTTGCCC
NF1_ex17	CCTCTTGGTTGTCAGTGCTTC	CTGTACCTTTGTTAGCCGGTACG
NF1_ex18	ATGTCTTCCACCCTTGACTCTC	GATGCCATGTGCTTTGAGGC
NF1_ex44	ACTTGCATGGACTGTGTTATTGG	AGCTCTTGGTTGCAGGGATG
NF1_ex46	GCTAGCTACCAAGATCACCATAGC	CAAGCGCTTGAGAACATACTATCC
NF1_ex50	AGGAGACTGTAAGAAGTTCATCCTG	CTGCTTGCCTCCATTAGTTGG
PTEN_ex1	CGCCACCAGCAGCTTCTG	CCAAACTACGGACATTTTCGCATCC
PTEN_ex6	ATGGCTACGACCCAGTTACC	GCTTCTTTAGCCCAATGAGTTGAAC
PTEN_ex7	GTTTGACAGTTAAAGGCATTTCCTGTG	TGCCAGAGTAAGCAAAACACCTG
ERCC4_ex8	GGCACAGGGAAACTAGGAGG	AACCCAGAAGCGGATGGATG
FANCA_ex4	TGCCACCAGTTTTATTGTGAGC	GTAACAACGGGCAGGTTTCC
FANCA_ex27	ATGCTCAGGCCATCCAGTTC	GTCCGAAAGCTGCGTAAACC
FANCA_ex33	CCTTGGGAAGGAGCAGAGTG	GCTGGTTTGGTGATGTGCTG
FANCA_ex35	GTTGGTCTCGCCTCTCTTCC	CCCTGAGATGGTAACACCCG
FANCD2_ex25	AGCAAGGACAGCTAGCTCCAG	CAGCTAGAGAAGCACACGGG
FANCG_ex10	GGTGAAGAGGTTGGGGATGG	GAGCTCACCAACTCAGGTCC
MLH1_ex2	GAAATGATGGTTGCTCTGCCC	GACTCTTCCATGAAGCGCAC
MSH2_ex3	AGGCTTCTCCTGGCAATCTC	TCCTAGGCCTGGAATCTCCTC
MSH2_ex6	GGGAGAAATGTACAGTTGAACATACGG	GTGGTATAATCATGTGGGTAACTGC
MSH2_ex11	GGATATGTTTCACGTAGTACACATTGC	ACCAAAAGCCAGGTGACATTC
MSH2_ex12	CTGTTGTCAGCTTTGCTCACG	ACGTTACCCCCACAAAGCC
MSH6_ex4	TGGGTCTCCCCTGAAGAGTC	AAGGCTGTATCCCATCGGTTC
PARP1_ex4	TATCCTTGCTTGTCGCTCCC	GAACCTGTAGGGCCTTTGGG
PARP1_ex8	GGTGGTTACCTCAAGGGAGAG	TCCACACCACCAGCAAGTAG
PMS2_ex11	ATGCTGTCTTCTAGCACTTCAGGTG	TCCCCTTCACTTTGCTGTGC
PRKDC_ex11	GAGACTAAGCAAAATGGAAGCAAAGG	CGCCCATGTTATTCAAGGGTCTAC
PRKDC_ex19	TGTTCTGACTAGCCTGTGCCTTG	GATTAAGGGCATGAGCCACCG
PRKDC_ex65	GCTTCTCTTTTCTCTCTCCTTTCCG	CCCTAGTGCTTCACAAGTACTACCTG
PRKDC_ex83	TTAGTGGGATCACAGCAGCC	ATGATGACTCTCGCCTTGCC
PRKDC_ex84	GGATGCTGCCATTTGTGCTC	GCTTGATCACGGGGACACTG
XPC_ex2	TTATGCCCCTGCCCATAAGC	CTTCCATGGACCCCAGTGAC
XPC_ex9	GCAGCGGCTCTGATTTTGAG	TCGCTGCACATTTTCTTGCC
XRCC2_ex3	TGGATAGACCGCGTCAATGG	TCCACATCACACAGTCGTCG
CCND1_ex5	CTGCCAGGAGCAGATCGAAG	GGAGAGGAGGGACTGTCAGG
CDKN2A_ex3	GCTTCCTTTCCGTCATGCCG	CGTGAGCTGAGGCAAGACC
IDH1_ex9	CGGTCTTCTGTTTGGGACAAGC	CCCTGGAATGACCCTGTTCC
RB1_ex23	GCTTCCACCAGGGTAGGTC	AGATTTTCTTCACCCCGCCC
RB1_ex20	CTCTGGGGGAAAGAAAAGAGTGG	GTAAGTAGGGAGGAGAGAAGGTGAAG
